# Use of Magtrace as an Alternative Approach in Staging the Axilla in Early-Stage Breast Cancer: A Single-Center Experience

**DOI:** 10.7759/cureus.75015

**Published:** 2024-12-03

**Authors:** Rami Oweis, Aditya Anil, Alexander Marinov, Tawfiq Hamati, Tahir Masudi

**Affiliations:** 1 Department of Breast Surgery, The Rotherham NHS Foundation Trust, Rotherham, GBR; 2 School of Medicine and Population Health, The University of Sheffield, Sheffield, GBR; 3 Department of General Surgery, Barnsley Hospital NHS Foundation Trust, Barnsley, GBR

**Keywords:** axilla, breast cancer, magtrace, sentinel lymph node biopsy, slnb

## Abstract

Background

The sentinel lymph node biopsy (SLNB) is the standard method used to determine the stage of breast cancer in patients with no clinical signs of axillary involvement. The current gold standard for the intraoperative assessment of the axilla involves the use of dual radioisotope and patent blue dye. However, researchers have been studying the use of superparamagnetic iron oxide Magtrace® (Endomagnetics Limited, Cambridge, United Kingdom) agents as an alternative to overcome the limitations of the standard SLNB technique.

Aim

The objective of this study was to assess the potential equivalency of the new SentiMag® (Endomagnetics Limited, Cambridge, United Kingdom) technique compared to the gold standard. The primary endpoints included the detection rate per patient and the number of lymph nodes retrieved.

Materials and methods

A single-center retrospective study was done in the Rotherham NHS Foundation Trust, UK, for patients who underwent SLNB from October 2021 to January 2022. Sixty patients were randomly selected and assigned into two arms: Magtrace and technetium-99m (99mTc). Categorical variables were compared using Fisher's exact test. Student's t-test was performed to compare continuous variables.

Results

There was a higher incidence of axillary seroma observed in the group of patients who underwent the Magtrace procedure. The average duration of the surgical procedure was longer for patients in the Magtrace arm compared to those in the 99mTc arm. The detection rate of SLNB was higher in the group of patients who received 99mTc. Both groups had a similar number of lymph nodes retrieved during the procedure. The length of hospital stay was comparable between the Magtrace and 99mTc groups.

Conclusions

Magtrace shows comparable practicality, surgical implementation, and risk of complications to standard practices. Additionally, it offers a potential cost benefit.

## Introduction

Sentinel lymph node biopsy (SLNB) is the established technique for staging clinically node-negative breast cancer. SLNB, first described by Giuliano et al. in 1994, has superseded the need for axillary lymph node dissection (ALND) as the standard procedure [1]. The minimally invasive nature of SLNB reduces the morbidity associated with ALND [2]. The use of a radiotracer in combination with blue dye has resulted in sentinel node detection rates of up to 97% [2]. Therefore, SLNB using a radioisotope with blue dye is the gold standard for intraoperative axillary node assessment [1,2].

Technetium-99m (99mTc), a metastable nuclear isomer of technetium-99, is the most commonly used radiotracer for SLNB [3]. Subareolar or peritumoral radioisotope dye injection is performed preoperatively [4]. 99mTc decays into its ground state through gamma emissions permitting the intraoperative assessment and identification of positive nodes using a gamma probe [3,5]. Blue dye also may be used to stain positive lymph nodes [6]. 99mTc is generally considered safe for both patients and staff; however, the radioactive nature of the isotope results in the strict regulation of its use, management, and disposal to prevent unintended contamination [6,7]. Furthermore, 99mTc is not easily accessible in most parts of the world, and the increased costs associated with the special handling required of the radiotracer limit the expansion of conventional SLNB [3-6].

Superparamagnetic iron oxide nanoparticles (SPIO) have been studied as an alternative to radioisotope tracers for SLNB [8]. The superparamagnetic properties of the nanoparticles allow for high spatial resolution and sensitivity through the detection of the net magnetization of the particles when an external magnetic field is applied [9]. Magtrace® (Endomagnetics Limited, Cambridge, United Kingdom) has been introduced as an SPIO-based tracer which can be detected using the SentiMag® handheld probe (Endomagnetics Limited, Cambridge, United Kingdom) [10]. The use of SPIO-based agents in staging the axilla, which has been studied in different trials, has shown non-inferiority to the 99mTc for axillary SLN detection in early-stage breast cancer [5,7-10].

Given the equivalence of SPIO-based tracers to the current gold standard, it may be preferential to the 99mTc-based radiotracer due to the logistical benefits associated with SPIO. Firstly, since SPIO-based tracers are not radioactive, there is no requirement for the special handling and disposal of the dye itself and any consumable equipment contaminated by the radioactive dye [11]. Furthermore, Magtrace® can be injected up to 30 days prior to the procedure, unlike the radiotracer which can only be injected up to a maximum of 24 hours prior to the procedure owing to the six-hour half-life of the isotope [12]. This allows for increased flexibility for the patient. Additionally, the increased buffer time allows for redundancy in case of operating list delays or cancellations which is not possible with the radiotracer [11-14].

The aim of this study was to evaluate the potential equivalency of the new Magtrace® and SentiMag® techniques in comparison to the "gold standard" technique using 99mTc-based radiotracer. The primary outcome was the node detection rate per patient and the number of lymph nodes retrieved. The secondary outcome was the postoperative length of stay and postoperative complication rates [15,16].

## Materials and methods

We conducted a retrospective cohort study at the Rotherham NHS Foundation Trust, UK. The study period was from October 1, 2021, to January 31, 2022. The study data was collected from physical and digital patient medical records. A master list of all adult female patients who underwent breast surgery with SLNB for invasive breast cancer was compiled. This was then stratified into two groups: the "control" group using the 99mTc radiotracer and the "intervention" group using Magtrace. Thirty patients were randomly assigned to each group using simple random sampling. Ethical approval was obtained from the Local Ethics Department of the Rotherham NHS Foundation Trust (approval number: R1339).

The inclusion criteria for this study were as follows: primary breast cancer stages T1-T3 and >18 years of age of any gender. Patients who underwent neoadjuvant chemotherapy were excluded as well as any metastatic disease at the diagnosis. Additional exclusion criteria were pregnancy, previous axillary surgery, radiation therapy to the axilla, or impaired axillary lymphatic function.

The primary endpoint for this study was the nodal detection rate. The number of nodes retrieved, postoperative complications, and the total length of stay were measured as the secondary outcomes. 

Categorical variables were compared using Fisher's exact test. Student's t-test was performed to compare continuous variables. A p-value of <0.05 was considered statistically significant. All statistical tests were performed using MATLAB version: 9.14.0 (R2023a) (The MathWorks Inc., Natick, Massachusetts, United States; https://www.mathworks.com).

## Results

Patient characteristics 

We analyzed a total of 60 patients, of which 30 received SLNB using Magtrace® and 30 received SLNB using 99mTc. The mean age of all patients was 64.25±11.6 (33-85) years. Between both groups, the mean ages were slightly dissimilar, but not statistically significant (Magtrace®: 66.9±11.8 vs. 99mTc: 61.6±11.1; p=0.077). The mean BMI of all patients was 28.8±6.2 (16-47). BMI was similar in both groups (Magtrace®: 29.8±6.7 vs. 99mTc: 27.9±5.8; p=0.189). Other patient characteristics such as tumor lateralization, tumor and nodal stage, tumor histopathology, and estrogen receptor (ER) and human epidermal growth factor receptor 2 (HER-2) status were similar and are shown in Table 1. 

**Table 1:** Clinical, demographic, and histopathological features NST: no special type; ILC: invasive lobular cancer; ER: estrogen receptor; HER-2: human epidermal growth factor receptor 2

	Magtrace (n=30)	Technetium (n=30)	P-value	T-value
	Mean (SD) or N (%)	Mean (SD) or N (%)		
Age	66.9 (11.8)	61.6 (11.1)	0.077	1.7936
BMI	29.8 (6.7)	27.9 (5.8)	0.189	1.1629
Lateralization			0.605	
Right	13 (43.3)	15 (50)		
Left	17 (57.6)	15 (50)		
Tumor stage			0.256	
pTis	0 (0)	3 (10)		
pT0	2 (6.7)	0 (0)		
pT1	17 (57.6)	14 (46.6)		
pT2	10 (33.3)	11 (36.7)		
pT3	1 (3.3)	1 (3.3)		
Missing	0 (0)	1 (3.3)		
Nodal stage			0.2326	
N0	25 (83.3)	20 (66.7)		
N1	5 (16.7)	9 (30)		
Missing	0 (0)	1 (3.3)		
Tumor histopathology			0.077	
NST	22 (73.3)	21 (70)		
ILC	7 (23.3)	3 (10)		
Other	1 (3.3)	6 (20)		
ER status			1	
Positive	26 (86.7)	25 (83.3)		
Negative	4 (13.3)	5 (16.7)		
HER-2 status			0.2542	
Positive	2 (6.7)	6 (13.3)		
Negative	28 (93.3)	24 (86.7)		

Intraoperative characteristics 

We found the total operative time matched for patients who only had wide local excision (WLE) with SLNB to be statistically significantly higher in the Magtrace® cohort than the 99mTc cohort (Magtrace®: 89.0±36.9 (30-146) minutes vs. 99mTc: 69.4±17.6 (36-101) minutes; p=0.012). We also found the surgery type between the two groups to be significantly variable with a p-value of 0.049. The breakdown of surgery type can be seen in Table 2. 

**Table 2:** Operative details and the number of LN retrieved SLNB: sentinel lymph node biopsy; LN: lymph node

	Magtrace (n=30)	Technetium (n=30)	P-value	T-value
	Mean (SD) or N (%)	Mean (SD) or N (%)		
Surgery type			0.049	
Breast-conserving surgery	21 (70)	28 (93.3)		
Mastectomy	7 (23.3)	2 (6.7)		
SLNB only	2 (6.7)	0 (0)		
Operative time	89 (36.9)	69.4 (17.6)	0.012	2.6116
Nodal detection			0.492	
Yes	28 (93.3)	30 (100)		
No	2 (6.7)	0 (0)		
LN retrieved	2.7 (1.49)	2.9 (1.17)	0.505	0.6743

Nodal detection was similar in both cohorts; however, the 99mTc cohort had a 100% nodal detection rate compared to 93.3% with Magtrace®. Nonetheless, the results were statistically comparable in our study. Additionally, lymph node retrieval and SLN detection were similar with both techniques. The detailed characteristics have been documented in Table 2.

Postoperative characteristics 

The median length of stay was identical in both groups at postoperative day 1 (p=1). We found an overall complication rate of 20% (n=12): eight complications in the Magtrace® group and four complications in the 99mTc group. Axillary seroma was the most common complication (n=10), with six axillary seromas developing in the Magtrace® cohort and four seromas developing in the 99mTc cohort. Additionally, there was one instance of breast superficial skin necrosis in the Magtrace® group. There was no statistical significance between complication rates in the two cohorts with a p-value of 0.333 as shown in Table 2.

## Discussion

Our study aimed to evaluate the role of Magtrace® at our district general hospital by comparing its efficacy with the established gold standard 99mTc radioisotope-based SLNB. This novel technique has already demonstrated its efficacy and equivalence in trial settings. However, our aim was to determine whether these results can be replicated in everyday practice. 

Our primary outcome measure was the nodal detection rate. We found no statistical significance in the nodal detection rate which supports the findings in published literature. The SentimagIC trial established Magtrace® as non-inferior to 99mTc radioisotope-based SLNB by undertaking nodal detection with Magtrace®, followed by confirmation using the dual tracer method. In this study, the dual tracer method identified nodes in 98.6% of patients, while the magnetic tracer identified nodes in 99.3% of patients [13]. Furthermore, there was a 100% concordance between dual tracer and magnetic tracer node detection [13]. Our study reflects similar findings with a 93.3% node detection rate in the Magtrace® arm and a 100% node detection rate in the 99mTc arm. 

The number of lymph nodes retrieved was similar in both cohorts. Nevertheless, SPIO-based tracers have been associated with higher nodal retrieval rates (p<0.0001) in a meta-analysis conducted by Karakatsanis et al. in 2016 [13]. It has been hypothesized that increased nodal retrieval may be associated with increased postoperative morbidity; however, no such association has been proven. Conflicting results have been reported in the literature following the 2016 meta-analysis with Shams et al. [15] also finding a significant association between magnetic tracers and an increased node retrieval rate, whereas the SMART study found no difference between their cohorts. 

Our study found the total operative time to be significantly higher in the Magtrace® cohort versus the 99mTc tracer group (89.0±36.9 minutes vs. 69.4±17.6 (36-101) minutes; p=0.012, respectively). As this was a retrospective study, we were unable to acquire data for lymph node detection time alone. Furthermore, due to there being a significant variation in the procedures performed between the groups, an accurate conclusion cannot be drawn in this regard due to the possible confounding effect. There is a relative paucity in the literature regarding the differences in the mean operative time between both techniques; however, Shams et al. [15] did report no difference in the mean operative time in their study. 

With an identical postoperative length of stay in both groups and no significant difference in postoperative complications, this was a secondary outcome measure that we had specified at the beginning of this study. Given the comparability of results between the study groups, within our study, the postoperative complication rate and postoperative length of stay associated with Magtrace® were equivalent to the established gold standard technique using 99mTc. 

The 99mTc-based tracer must be administered 3-24 hours preceding the procedure owing to its short half-life of <6 hours. Furthermore, the administration of this must take place within a nuclear medicine department. Careful coordination between the nuclear medicine department and operating department is essential, to ensure adequate 99mTc during the procedure. 

SPIO-based tracers afford both clinician and patient flexibility, unlike the 99mTc-based tracer as Magtrace® can be administered on the operating table not only 20 minutes prior to the procedure but also up to 30 days prior to the procedure as licensed by the National Institute for Health and Care Excellence (NICE). This not only mitigates the unnecessary radiation exposure to the patient in the event of the cancellation of a procedure but allows for patients to have adequate time and flexibility prior to the procedure, unlike the restrictive timing necessitated by 99mTc-based tracer injection. 

At our institution, 22 patients received the Magtrace® injection on the operating table. This obviated the need for the patient to come into the hospital prior to the procedure for the tracer injection. As a result, the increased operative time observed with the Magtrace® cohort may not be as significant as the pre-surgical procedure was not required. Furthermore, given that clinicians must wait 20 minutes after the administration of Magtrace® to start the procedure, this may explain the significantly longer operative time seen with the Magtrace® cohort.

The main limitation of this study is its small sample size and retrospective design. We believe that including a larger number of patients would enhance the significance of the findings.

## Conclusions

Our study demonstrates that Magtrace® can successfully be implemented in our institution and similar district general hospitals with outcomes equivalent to the current established gold standard practice of using 99mTc-based tracers to facilitate SLNB in breast cancer. Furthermore, not only were detection rates equivalent in the Magtrace® cohort, but postoperative length of stay and complication rates were similar in both groups as well. Therefore, Magtrace® can be feasibly and safely implemented in a district general setting with equivalent efficacy. 
